# Comparison of neurocognitive changes among newly diagnosed tuberculosis patients with and without dysglycaemia

**DOI:** 10.1186/s12888-020-02570-8

**Published:** 2020-04-03

**Authors:** Ernest Yorke, Vincent Boima, Ida Dzifa Dey, Vincent Ganu, Norah Nkornu, Kelvin Samuel Acquaye, C. Charles Mate-Kole

**Affiliations:** 1 Department of Medicine & Therapeutics, School of Medicine & Dentistry, College of Health Sciences, University of Ghana, Legon, Accra Ghana; 2 Department of Medicine, Korle-Bu Teaching Hospital, Accra, Ghana; 3 Department of Psychology, School of Social Sciences, College of Humanities, University of Ghana, Accra, Ghana; 4 Department of Social and Behavioural Sciences, School of Public Health, University of Ghana, Accra, Ghana; 5 Department of Psychiatry, School of Medicine & Dentistry, College of Health Sciences, Korle-Bu, Accra, Ghana; 6 Centre for Ageing Studies, College of Humanities, University of Ghana, Accra, Ghana

**Keywords:** Tuberculosis, Smear positive, Dysglycaemia, Neuropsychological disorders

## Abstract

**Background:**

Diabetes often occurs together with tuberculosis (TB) and both may affect each other negatively. Diabetes may be associated with neurocognitive dysfunctioning in affected patients and may negatively impact treatment adherence and outcomes. This study compared the neurocognitive status between newly diagnosed smear positive tuberculosis patients with dysglycaemia and those with normoglycaemia.

**Methods:**

The current study was a cross-sectional study involving one hundred and forty-six (146) newly diagnosed smear positive TB patients. Oral glucose tolerance test (OGTT) was performed and the results were categorized as either normoglycaemia, impaired glucose tolerance (IGT), impaired fasting glucose (IFG) or diabetes.

Neurocognitive functioning among study participants was assessed at the time of TB diagnosis using Cognitive Failure Questionnaire (CFQ), Montreal Cognitive Assessment tool (MoCA), California Verbal Learning Test (CVLT), Brief Symptom Inventory (BSI) and the Spitzer Quality of Life Index (QLI).

**Results:**

The mean age of the participants (*n* = 146) was 38.7 years with 78.8% being males and 21.2% females. Using the fasting blood glucose test, the prevalence of impaired fasting glucose and diabetes were 5.5 and 3.4% respectively, both representing a total of 13 out of the 146 participants; whilst the prevalence of impaired glucose tolerance and diabetes using 2-h post-glucose values were 28.8 and 11.6% respectively, both representing a total of 59 out of the 146 participants.

There were no significant differences in the mean scores on the neurocognitive measures between the dysglaycaemia and normoglycamic groups using fasting plasma glucose (FPG). However, there were significant differences in the mean scores between the dysglycaemia and normal groups using 2-h postprandial (2HPP) glucose values on Phobic Anxiety (Normal, Mean = 0.38 ± 0.603; dysglycaemia, Mean = 0.23 ± 0.356; *p* = 0.045), and Montreal Cognitive Assessment (MoCA) scores (17.26 ± 5.981 vs. 15.04 ± 5.834, *p* = 0.037).

**Conclusion:**

Newly diagnosed smear positive patients with dysglycaemia were associated with significantly lower mean cognitive scores and scores on phobic anxiety than those with normoglyacaemia. The latter finding must be further explored.

## Background

A lot of effort continues to be made to reduce the worldwide incidence, morbidity and mortality associated with tuberculosis (TB); however it continues to be a deadly communicable disease. In 2015, 10.4 million new cases and 1.4 million deaths due to TB were estimated to have occurred by World Health Organisation (WHO) [1]. Many reviews and meta-analysis have reported that diabetes increases TB risk by between 1.5 to 7.8 fold [2, 3]. Since developing countries have the greatest burden of TB, the expected increase in the prevalence of type 2 diabetes in these parts of the world would increase the impact of diabetes on TB [1, 4]. Negative impact of comorbid diabetes on TB include poor treatment outcomes [2], more severe disease and re-activation of dormant tuberculosis foci [5].

Tuberculosis (TB) like many chronic diseases is associated with a high burden of psychological disorders which may have a negative impact on the psychological health, treatment adherence and outcomes [6].

Common psychological disorders among TB patients especially around the time of diagnosis include depression, anxiety, in addition to poor quality of life [6]. The reasons for these changes include physiological and physical impact of the disease, stigma and isolation, effects of medications and rigours of keeping appointment and other negative social impact including loss of income [6]. These problems may affect self-esteem and attitudes towards disease management including compliance to medications and eventually lead to poor disease outcome [6]. The association of cognitive impairment with pulmonary TB alone does not appear to be strong, although this may be the case among patients with systemic spread [7–9]. However, cognitive dysfunction has been strongly associated with diabetes, which may impair self-management activities in many patients [10–12].

The negative impact of diabetes on TB, including potential cognitive impairment, coupled with the expected increases in the prevalence of both diseases is likely to portend bad prognosis in the management of TB patients [1, 2, 4–6]. Unfortunately, developing nations have limited healthcare budgets and resources to deal with the expected negative reciprocal impact of diabetes and TB on the affected population.

Despite the plethora of information on the potential interaction and effect of diabetes on cognitive function in general population, there is minimal published data about the potential cognitive impact of diabetes and dysglycaemia on cognition among TB patients [2, 3, 5, 6]. This study examined and compared the neurocognitive functioning among newly diagnosed smear positive tuberculosis patients at time of diagnosis with dysglycaemia and those without.

## Methods

### Study site and design

We conducted a hospital based cross-sectional study at the Chest Clinic of the Korle-Bu Teaching Hospital, which is a tertiary care facility and the largest hospital in Ghana. The Korle-Bu Chest clinic is the main referral centre in southern Ghana for TB cases and other respiratory conditions.

### Participants and recruitment

Inclusion criteria included TB patients who were newly diagnosed smear positive cases and were either initially diagnosed or referred for treatment at the Chest Clinic, 18 years and above with no previous history of TB treatment. None of the patients had known pre-existing diabetes. Excluded participants were those aged below 18 years, those with previous history of TB treatment, patients with smear negative TB, extra pulmonary TB, those with previous history of psychological, psychiatric or neurological disorders or those who refused consent. We used a convenient sampling method to recruit about 3–4 participants on a weekly basis (week days only) who met the included criteria. A total of 160 participants were recruited over a 12-month period.

### Measurements

At enrolment, data on demographic and anthropometric characteristics such as body mass index (BMI), waist circumference (WC), hip circumference (HC) and waist-hip-ratio (WHR) as well as medical history were collected using standardized questionnaire.

BMI (calculated) was categorized as obese, overweight, normal and underweight with defined values of 30.0 or more, 25–29.9, 18.5–24.9-and less than 18.5 (Kg/m^2^) respectively [13].

Microscopy using Ziehl-Neelsen (ZN) stain was used to determine the presence of acid fact bacilli (AFBs) [14]; whilst plasma glucose levels were determined using a 75 g oral glucose tolerance test (OGTT). After an overnight fast (8 h), ten millilitres (ml) of fasting blood sample was taken into fluoridated blood sample tubes and centrifuged within 15 min of blood draw after being kept on ice [15]. After 2 h of the administration of 75 g glucose in 250 ml water a second blood sample was taken into fluoride tubes for glucose determination. A commercial glucose oxidase reagent kits and controls (Diasys, GmBH, Germany) was used to determine plasma glucose; which were then categorized into normal glucose levels, impaired glucose tolerance (IGT), impaired fasting glucose (IFG) and diabetes.

A FPG and 2HPP values of 6.1–6.9 mmol/l and 7.8–11 mmol/l diagnosed impaired fasting and glucose tolerance respectively; whilst FPG and 2HPP are > 7 mmol/l and > 11.1 mmol/l respectively or a person on regular medication for diabetes was labelled a diabetic. Normal plasma glucose values were defined by FPG and 2HPP values below 6.1 mmol/l and 7.8 mmol/l respectively [16]. Using fasting blood glucose values, dysglycaemia was defined as those with IFG and/or diabetes whilst using 2HPP values, it was defined as those with IGT and/or diabetes. All patients diagnosed with any form of dysglycaemia were referred to diabetes specialists for continued care [16].

Neuropsychological measurement tools include the Cognitive Failure Questionnaire (CFQ) [17], Montreal Cognitive Assessment tool (MoCA) [18], California Verbal Learning Test (CVLT) [19], Brief Symptom Inventory (BSI) [20] and the Spitzer Quality of Life Index (QLI) [21]. BSI assesses various psychological domains including depression, anxiety, somatization, paranoid ideation, phobia anxiety, obsessive compulsive behaviour, and interpersonal sensitivity [20]. For the purposes of this study only depression, anxiety, somatization, phobia anxiety as well as the Global severity index (GSI) of the BSI [22] were analysed. GSI of the BSI is calculated using the sums for the nine symptom dimensions plus the four additional items not included in any of the dimension scores, and dividing by the total number of items to which the individual responded. If no items were skipped the GSI will be the mean for all 53 items. The mean scores of the various domains including GSI are interpreted by comparison to age-appropriate norms [20]. The Spitzer Quality of Life Index (QLI) [21] rates patient’s well-being in the areas of health, activity, daily living, outlook and support. The QLI yields a score that ranges from a high of 10 to a low of 0. The higher one’s score on this measure, the better his or her quality of life. Cognitive Failure Questionnaire (CFQ) [17] assesses self-reported cognitive lapses in perception, memory and misdirected action. The total score on the scale is obtained by adding up the ratings of the 25 individual items, yielding a score from 0 to 100. Score ranges between 0 and 50 is within the average scores and reflect normal functioning. Scores above 50 reflects clinically relevant problems. Montreal Cognitive Assessment tool (MoCA) [18] is a widely used screening assessment for detecting cognitive impairment. MoCA scores range between 0 and 30. A score of 26 or over is considered to be normal.

CVLT is a measure of verbal learning and memory, which demonstrates sensitivity to a range of clinical conditions such as recall, cognition and encoding [19]. CVLT short form was used; It consists 9 items and there are no cut-offs for normality, however the higher the scores (the number of items one can recall) the better the memory.

### Statistical analysis

The Statistical software Stata version 15 was used for the analysis of data of 146 patients with complete OGTT information. It was imported from Microsoft Excel version 2010 following an initial cleaning.

Socio-demographic, anthropometric, medical history and glycaemic variables were summarised descriptively as means and standard deviations, frequencies and percentages, For the purposes of analysis, dysglycaemia (abnormal glucose level) was defined to include the combined prevalence of impaired fasting glucose and diabetes (using fasting glucose values) or those with impaired glucose tolerance and diabetes (using 2-h postprandial values). Mean baseline scores of participants on neurocognitive measures tools were compared using an independent T-test at baseline between those with dysglycaemia and those without using both FBS and 2HPP values. The statistical significance levels were set at 5%.

## Results

One hundred and forty-six (146) participants (78.8% males and 21.2% females) with a mean age of 38.7 years were involved in the study. Majority (51.37%) of our participants were single with 36.99% having education up to Junior High School (JHS) level (Table 1). Fasting blood glucose tests results showed 5.48% had impaired glucose levels whilst 3.42% had diabetes (Table 1). 2-h postprandial results showed 28.77% had impaired glucose levels whilst 11.64% had diabetes (Table 1).

**Table 1:** Background Characteristics of newly diagnosed smear positive tuberculosis patients at the Chest Clinic of the Korle-Bu Teaching hospital in Accra, Ghana, 2017

	Frequency	Percentage
**Age** (mean ± SD)	38.70 ± 13.97	
**Sex**		
Female	31	21.20
Male	115	78.80
**Highest educational level**		
Tertiary	23	15.75
O-Level/A-level/SHS	32	21.92
Middle School/ JHS	54	36.99
Primary	14	9.59
None	13	8.90
Other	10	6.85
**Marital Status**		
Single	75	51.37
Married	62	42.47
Separated/Divorced	9	6.16
**Employment status**		
Unemployed	38	26.03
Employed	108	73.97
**Alcohol intake**		
Yes	22	15.07
No	124	84.93
**Smoking**		
Yes	10	6.85
No	136	93.15
**Weight** (mean ± SD)	52.74 ± 8.94	
**Height** (mean ± SD)	1.68 ± 0.08	
**BMI**		
Above 30 (Obese)	1	0.68
25–29.9 (Overweight)	2	1.37
18.5–24.9 (Normal)	40	27.4
Below 18.5 (Underweight)	103	70.55
Mean ± SD	18.49 ± 3.00	
**2-HPP Glucose (mmol/L)**		
Diabetes (> 11.1)	17	11.64
Impaired (7.8–11.1)	42	28.77
Normal (< 7.8)	87	59.59
Mean ± SD	8.35 ± 3.44	
**Fasting Plasma Glucose (mmol/L)**		
Diabetes (> 7.1)	5	3.42
Impaired (6.1–7)	8	5.48
Normal (< 6.1)	133	91.1
Mean ± SD	5.12 ± 1.52	

SD Standard deviation, *DBP* Diastolic Blood pressure, *SBP* Systolic Blood Pressure, *2HPP* 2-h postprandial

Information on the prevalence of diabetes and dysglycaemia in this study shown in Table 1 has been published earlier and discussed extensively [23].

### Mean scores on neuropsychological testing

The independent t-test conducted to compare the differences in the scores on neurocognitive tests (CFQ, MOCA, and CVLT) between dysglycaemia and those with normal glucose, using FBS showed no significant differences between the two groups.

However, on the behavioural measures there were significant differences between those with dysglycaemia and those without using 2HPP scores, and the scores of Phobic Anxiety t (150) = 2.025, *p* = 0.045. Participants’ with dysglycaemia had lower scores compared to those who had normal glucose values (Normal, Mean = 0.38 ± 0.603; dysglycaemia, Mean = 0.23 ± 0.356), Table 2.

**Table 2:** Summary of Independent t-test for 2HPP and neuropsychological measures among newly diagnosed smear positive tuberculosis patients at the Chest Clinic of the Korle-Bu Teaching hospital in Accra, Ghana, 2017

	2HPP	n	M	SD	t	df	p
Cognitive Failures (CFQ)	Normal	93	19.23	13.20	1.25	152	.202
	Abnormal	61	16.61	11.86			
Somatisation	Normal	92	1.27	.81	−.38	152	.704
	Abnormal	62	1.32	.77			
Anxiety	Normal	92	.55	.66	1.53	152	.106
	Abnormal	62	.41	.48			
Depression	Normal	91	.52	.59	.68	151	.477
	Abnormal	62	.46	.45			
Phobic Anxiety	Normal	92	.38	.60	2.03	149.7	.045
	Abnormal	62	.23	.36			
Perceived Psychological Distress (BSI-GSI)	Normal	92	.65	.47	1.21	151.8	.229
	Abnormal	62	.58	.30			
Total Free Recall	Normal	70	20.13	5.83	.87	105	.353
	Abnormal	37	19.16	4.65			
Short Delay Free Recall	Normal	70	5.47	1.96	1.61	105	.120
	Abnormal	37	2.12	1.61			
Long Delay Free Recall	Normal	68	4.76	2.02	1.03	102	.307
	Abnormal	36	4.33	2.04			
Cognitive Impairment (MOCA)	Normal	84	17.26	5.98	2.10	132	.037
	Abnormal	50	15.04	5.83			
Quality of Life	Normal	93	7.20	1.90	1.30	153	.197
	Abnormal	62	6.82	1.59			

*p* < .05 (2-tailed), Note: *M* Mean, *SD* Standard deviation, *df* Degree of freedom, *BSI* Brief Symptom Inventory, *GSI* Global severity index, *MOCA* Montreal Cognitive Assessment, *Abnormal* Dysglyacaemia

The scores on the Montreal Cognitive Assessment (MOCA) tool showed a significant difference in the mean scores between those with normal vs. dysglycaemia, t (105) =2.112, *p* = 0.037. Those with dysglycaemia had lower mean scores (Mean = 15.04 ± 5.834) compared with those with normal glucose (Mean = 17.26 ± 5.981). There were no significant differences in the rest of the mean scores for the other tests (Table 2).

## Discussion

In this study, using fasting glucose values, the prevalence of impaired fasting glucose and diabetes were 5.5 and 3.4% respectively. Using 2-h post-glucose, the prevalence of impaired glucose tolerance and diabetes were 28.8 and 11.6% respectively. These prevalence findings have already been published and discussed extensively [23] with similar results compared to other studies [24–26]. Significantly, most of the patients (about 70%) were underweight. TB patients tend to come from low socioeconomic background and generally malnourished and underweight [27]. Weight loss among TB patients may also be contributed to by the inflammatory response to infection [28, 29] as well as nausea, loss of appetite and vomiting.

### Diabetes and cognition decline among TB patients

The 2-h post-glucose values revealed that TB patients with dysglycaemia had a significant lower mean score than those with normal glucose values on MOCA. This implies that dysglycaemia is associated with poorer cognitive functioning compared to those with normal glucose levels among TB patients. This finding supports the conclusions from other studies that diabetes predisposes to cognitive decline both in humans and animal models [12, 30–33], and this finding also holds true for our study cohort who had TB and dysglycaemia. Cognitive decline may negatively affect compliance to the strict requirements of TB treatment including adherence to medications which can potentially worsen disease outcomes [34].

Whiles the pathophysiology of cognitive dysfunction in diabetes mellitus is putative; hyperglycaemia, vascular disease, hypoglycaemia, and insulin resistance are thought to play important roles [35]. Altered cerebral insulin and glucose homoeostasis may lead to widespread brain microangiopathy [36, 37]. Insulin resistance, a feature of diabetes and prediabetes, increases the formation of Advanced Glycation End-products (AGE) leading to the overproduction of Reactive Oxygen Species (ROS) which accelerate biological aging and subsequent cognitive decline [38]. The recognition of cognitive impairment in the dysglycaemia group in a relatively younger patient cohort in our study (average age 38.7 years) might suggest that cognitive impairment that occurs in patients with dysglycaemia and diabetes in TB patients probably occurs early on in the disease. This was supported by a study by Ruis et al. who found out that early on in the course of type 2 diabetes, there is modest cognitive decline [39].

The influence of pulmonary TB alone on cognitive decline does not appear to be strong. A study published in India in 2015 showed that cognitive impairment was significantly higher among HIV patients with Pulmonary Tuberculosis than in patients with HIV infection alone [40]. In the setting of non-clinically obvious systemic spread, especially among HIV patients, PTB may impact on neurocognitive function [41, 42] through granulomatous meningeal inflammation with the formation of exudate and adhesions, obliterative vasculitis, encephalitis or myelitis or hydrocephalus [7–9].

### Prediabetes and cognitive decline

The dysglycaemia group included TB patients with prediabetes who scored significant lower scores on cognitive tests. In a recent study published in 2017 [43], prediabetes was associated with lower performance in memory in middle age and but a less steep decline in memory over the follow-up period [43]. Also, the Confucius Hometown Aging Project, which was a cross-sectional study of 1528 participants conducted in Shandong province in China, examined the association of diabetes and prediabetes with cognitive impairment and depression among Chinese elderly people aged ≥60 years. Cognitive impairment and depression was found in 24.7 and 20.3% subjects respectively. The multiple adjusted odds ratio (OR) of cognitive impairment was 1.61 for prediabetes, 1.38 for diabetes, and 1.43 for having either prediabetes or diabetes [44]. .Another study found modest cognitive decline already present at the early stage of type 2 diabetes [39]. The results of this study are preliminary and our future study will employ more neuropsychological measures and larger numbers to assess various domains of cognition, and compare any differences between those with overt diabetes and prediabetes.

### Phobic anxiety

Our study revealed that participants with dysglycaemia had significantly lower scores on Phobic Anxiety compared to those who had normal glucose levels. This means that participants with dysglycemia reported less phobic anxiety compared to those with normal glucose levels. This finding however differs from other publications, which found higher anxiety related symptoms including phobic anxiety associated with diabetes [45–47]. Differences in participant characteristics such as the relatively young age of subjects and a different setting of the study may account for the observed differences in the findings. This must further be explored in subsequent studies.

### Future research

Future research must include extensive neuropsychological measures to assess various cognitive domains and establish the severity and degree of cognitive decline that may impact treatment outcomes among TB patients. Also since social, demographic and sanitary variables are often implicated in psychological issues concerning TB patients, these factors could be explored in the future studies as moderators/modulators of neurocognitive function as well.

## Conclusion

Our study is unique in examining the influence of dysglyacaemia on cognitive performance among young adults with TB. Newly diagnosed smear positive TB patients with dysglycaemia was associated with statistically significantly lower mean cognitive scores than those with normal glucose values. Also, patients with abnormal glucose values was associated with statistically lower mean scores on phobic anxiety as compared to those who were normoglycaemic; and this finding must be further explored.

The study findings have implications TB management. Among newly diagnosed smear positive patients, neuropsychological tests must be performed at diagnosis to ascertain the presence or otherwise of any neuropsychological deficits. Any deficits identified should be promptly treated to reduce its impact on the patient as well to improve treatment outcomes. It is hoped that this will spur increased interest and research on the impact of dysglycaemia on the cognitive function on TB patients and its impact on treatment outcomes.

## Data Availability

The datasets used and/or analysed during the current study are available from the corresponding author on reasonable request.

## Abbreviations

AFB: Acid fact bacilli
BMI: Body mass index
BP: Blood pressure
BSI: Brief Symptom Inventory
CFQ: Cognitive Failure Questionnaire
CI: Confidence Interval
CVLT: California Verbal Learning Test
FPG: Fasting plasma glucose
IGT: Impaired glucose tolerance
MDR: Multidrug resistant tuberculosis
MOCA: Montreal Cognitive Assessment tool
OGTT: Oral glucose tolerance test
OR: Odds ratio
SD: Standard deviation
QLI: Spitzer Quality of Life Index
TB: Tuberculosis
WHO: World Health Organisation
ZN: Zeihl Neelson
2HPP: 2-Hour Post-prandial glucose
